# *Arthrospira platensis* Extract: A Non-Invasive Strategy to Obtain Adjunct Attenuated Cultures

**DOI:** 10.3390/foods10030588

**Published:** 2021-03-11

**Authors:** Elena Bancalari, Francesco Martelli, Benedetta Bottari, Erasmo Neviani, Monica Gatti

**Affiliations:** Department of Food and Drug, University of Parma, Parco Area delle Scienze 49/A, 43124 Parma, Italy; francesco.martelli@unipr.it (F.M.); benedetta.bottari@unipr.it (B.B.); erasmo.neviani@unipr.it (E.N.); monica.gatti@unipr.it (M.G.)

**Keywords:** spirulina extract, attenuated cultures for cheese production, impedance microbiology, fluorescence microscopy, co-culturing, lactic acid bacteria, *Lacticaseibacillus*, NSLAB

## Abstract

This study aims at proposing the use of *Arthrospira platensis,* commonly known as Spirulina, extract as a non-invasive method to attenuate the growth rate of non-starter adjunct cultures, thus preventing the over-acidification that may occur during cheese manufacturing. A preliminary screening using four different concentrations (0.20%, 0.30%, 0.50%, and 0.70%) of *A. platensis* extract and four starter and three non-starter lactic acid bacteria strains was performed by impedometric analysis. This allowed us to select one starter and one non-starter strain to be used in the in vitro simulation of a co-culture in milk with the best antimicrobial concentration (0.3%). The growth dynamics of the two selected strains, starter *Lactococcus lactis* 1426 and non-sarter *Lacticaseibacillus rhamnosus* 1473, co-cultured for 120 h was monitored by three different approaches: (i) plate counting on M17, for the enumeration of lactococci, and MRS for lactobacilli; (ii) fluorescence microscopic counting of viable and non-viable coccoid *Lactococcus lactis* 1426 and rod-shaped *Lacticaseibacillus rhamnosus* 1473 cells; (iii) the overall estimation of co-culture growth behavior by impedometric parameters Lag, Rate, and yEnd. All the data obtained from the in vitro simulation were in agreement, revealing that a slowdown of non-starter growth occurred, while the starter strain was not affected, or slightly stimulated, from the antimicrobial presence. In particular, the growth of *Lb. rhamnosus* 1473 was delayed without adversely compromise the cells’ integrity, connected with metabolic functions, showing a great potential for use in cheese production.

## 1. Introduction

Non starter lactic acid bacteria (NSLAB), mainly belonging to the *Lacticaseibacillus* genus [1], predominate during the ripening of several cheese varieties and are known to be responsible for the development of appreciated organoleptic characteristics, as well as, in some cases, of defects [2]. As an example, the presence of adventitious NSLAB from milk in raw milk cheeses could introduce a variable in the ripening process that cannot be easily controlled, bringing about possible fluctuations in the characteristics of the final product [3,4]. This variability could be minimized by adding selected adjunct cultures and controlling their development. To this aim, as well as to enhance the flavor and sensorial characteristics of ripened cheeses, the use of non-starter as adjunct cultures has gained increasing attention during the last few years [2]. This kind of culture has also been used to enhance the sensorial characteristics of pasteurized or sterilized milk cheeses or the flavour of low-fat cheeses [5,6]. Unfortunately, the main drawback in the use of these cultures is the possible over-acidification due to their rapid growth, particularly during curd acidification, which could lead to changes in both the texture and sensory characteristics of the cheeses [5,6,7]. In fact, the production of an excess of lactic acid might negatively influence the final cheese flavour and texture [5]. To avoid this, a strategy developed throughout the years is the use of non-starter adjunct strains in form of attenuated cultures. These are LAB that do not have the capacity to grow and produce lactic acid during the first stages of cheese making and thus do not compete with the activity of the main starter. On the contrary, they accelerate and improve flavour and texture development during cheese ripening without affecting the characteristics of cheese by lowering the pH and changing the rheology of the product. To date, several methods have been used to achieve cultures attenuation, including heat treatment, freezing and thawing, freeze or spray-drying, lysozyme addition, the use of solvents, and high-pressure treatment [7]. More recently, sonication has also been proposed for the attenuation of adjuncts cultures of mesophilic NSLAB used in Italian Caciotta-type cheese making [5]. The desired result of using such above-mentioned methods is the achievement of a certain degree of cell lysis with the consequent release of intracellular enzymes in the matrix [8,9]. It is known that bacterial intracellular enzymes can be responsible for the important transformation of different milk components that lead to the formation of peculiar aroma during cheese ripening [10]. However, the attenuation of adjunct cultures should avoid adversely compromising their viability and functional metabolisms [11]. In this frame, a step forward in the obtainment of attenuated non-starter adjunct cultures could be the use of a non-invasive method to slow the cell growth rate while avoiding their disruption. To this aim, we propose the use of specific concentrations of *Arthrospira platensis,* commonly known as spirulina, extract. The antimicrobial activity of this extract has been previously studied against food pathogenic and food alterative bacteria, proving its high efficacy [12,13]. In the present study, the ability of different concentrations of *A. platensis* extract to modulate the growth of starter (SLAB) and NSLAB lactic acid bacteria was studied. By means of impedometric analysis and the fluorescent microscopic evaluation of cells’ viability, it was possible to observe the impact of *A. platensis* extract on the studied strains, suggesting the most suitable balance among SLAB, NSLAB, and *A. platensis* extract for possible application in cheesemaking.

## 2. Materials and Methods 

### 2.1. Arthrospira Platensis Extraction Process

The *A. platensis* powder used in the present study was kindly provided by S.a.Ba.r. (Novellara, Emilia-Romagna Region, Italy). The extraction process was performed foowing the protocol aready described by Martelli et al. (2020) [14]. 

After a double extraction followed by filtration (11 µm filter papers, 150 mm ø, Whatman, Schleicher & Schuell, Maidstone, GB), the extract was dried and then supended in sterile water before being stored at −80 °C until use. 

### 2.2. Bacterial Strains and Culture Conditions 

Four strains commonly used as starters in milk fermentation, such as *Streptococcus thermophilus* (2) and *Lactococcus lactis* (2), and three non starter strains *Lacticaseibacillus casei*, *Lacticaseibacillus paracasei*, and *Lacticaseibacillus rhamnosus*,belonging to the formerly known Lactobacillus casei group [1] were chosen for the study (Table 1).

The strains belonging to the collection of the Food Microbiology group of the Food and Drug Department (University of Parma, Italy), were previously isolated from different food matrixes and identified by 16S rRNA sequencing (Table 1). 

The strains, were maintained as frozen stock cultures at −80 °C in M17 (Oxoid, Ltd., Basingstoke, United Kingdom) broth for *Lactococcus* spp. and MRS (Oxoid, Ltd., Basingstoke, United Kingdom) broth for *Lactobacillus* spp, containing 20% (*v*/*v*) glycerol. The strains were revitalized in the appropriate medium by two overnight sub-culturing 2% (*v*/*v*) at 37 °C for *Lacticaseibacillus* strains, 30 °C for *L. lactis*, and 42 °C for *S. thermophilus* (Table 1). 

### 2.3. Experimental Design

**Figure 1:**
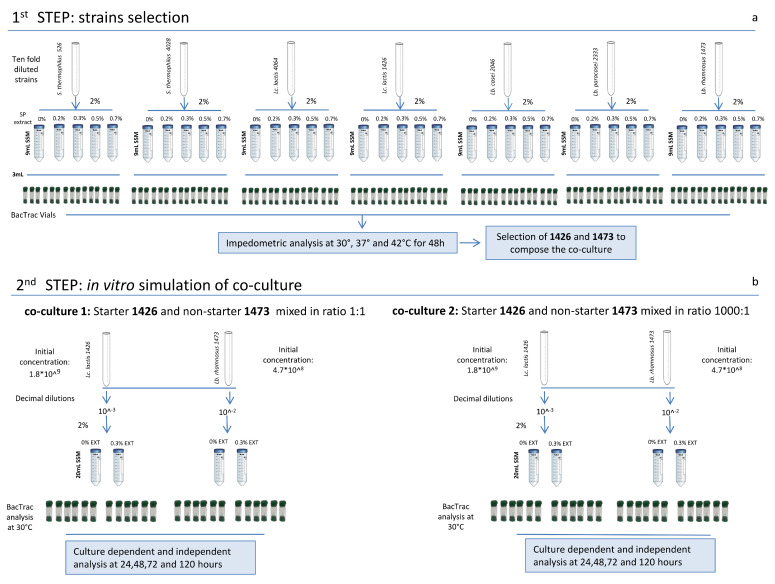
Experimental design. In the figure (**a**) the scheme of the strain selection precudure is reported, and (**b**) the schematic representation of the in vitro simulation of co-culture is reported.

The experimental design was divided into two main parts. At first, all the strains (Table 1), after revitalization, were tested for their ability to grow in milk with and without the addition of four different concentrations (0.20, 0.30, 0.50, and 0.70%) of *A. platensis* extract (Figure 1a). This step was crucial for the second part of the experiment, which consisted of the selection of two strains, one starter (SLAB) and one non-starter (NSLAB), to be used for the in vitro simulation of a co-culture. The fermentation was carried out for 120 h, performing culture dependent and independent analyses at each samping time (Figure 1b).

### 2.4. Single Strain Growth Condition and Analysis

After two sub-culturing step in MRS and M17 (Table 1), the strains were sub-cultured twice and incubated overnight at their optimum temperature, as reported in (Table 1), in (2% *v*/*v*) skim milk powder (SSM) (Oxoid Ltd.). SSM was reconstituted to 10% (*w*/*v*) and pasteurized in an autoclave at 90 °C for 10 min before use. *A. platensis* extract was added to the SSM to reach final concentrations (*v*/*v*) of (a) 0.20%, (b) 0.30%, (c) 0.50%, d) 0.70%. A negative control sample without any addition of extract was also considered as e) 0% (Figure 1). After the last sub-culturing step in SSM, each bacterial culture was ten-fold diluted in Ringer’s solution (Oxoid) and used to inoculate 2% (*v*/*v*) 9 mL of SSM supplemented, respectively and separately, with all the different concentrations of *A. platensis* extract (Figure 1). A total of 3 mL of inoculated SSM was transferred into sterilized BacTrac 4300^®^ vials (SY-LAB, Neupurkersdorf, Austria), and incubated at the optimal growth temperature of each strain (Table 1). All the analyses were carried out in triplicate and monitored for 24 h by measuring the impedometric signal every 10 min by the BacTrac 4300^®^ Microbiological Analyzer (SY-LAB, Neupurkersdorf, Austria). 

### 2.5. In Vitro Simulation of Selected SLAB and NSLAB 

The starter strain *L. lactis* 1426 and the non-starter strain *Lb. rhamnosus* 1473 were selected and used to prepare the co-culture of (1) *L. lactis* 1426 and *Lb. rhamnosus* 1473, added to SSM in a ratio of 1:1, and the co-culture of (2) *L. lactis* 1426 and *Lb. rhamnosus* 1473, added to SSM in a ratio of 1000:1. The initial concentration of the two strains was estimated by plate count as 4.7 × 10^8^ for *Lb. rhamnosus* 1473 and 1.8 × 10^9^ for *L. lactis* 1426. These were differently diluted to reach the same inoculum level to be added in SSM (Figure 1b). Both co-cultures 1 and 2 were inoculated in SSM without antimicrobial addition (0%), named Control (C), and with the addition of 0.3% of antimicrobial (A), and then analysed continuously for 120 h at 30 °C by a BacTrac 4300^®^ Microbiological Analyzer system. After 24, 48, 72, and 120 h of incubation, samples were taken from the vials for the pH measurement and culture dependent (viable count) and independent (Fluorescence microscopy) analyses (Figure 1b).

### 2.6. Impedometric Measurement

Impedance analyses were performed for single strains and co-cultures by means of the BacTrac 4300^®^ Microbiological Analyzer system. The M% values were measured and recorded every 10 min for 48 h [15,17]. Each experiment was replicated twice and each analytical variable was measured in triplicate. The results of the impedometric analysis were analyzed, as previously reported by Bancalari et al. (2016) [15].

### 2.7. pH Measurement

After 24, 48, 72, and 120 h of incubation pH was measured by means of a pH meter (Beckman Instrument mod Φ350, Furlenton, CA, USA) and a glass electrode (Hamilton, Bonaduz, Switzerland). 

### 2.8. Culture-Dependent Viable Counts of the Co-Cultures 

After 24, 48, 72, and 120 h of incubation of the two co-cultures (Figure 1), aliquots of the fermented SSMs were aseptically taken, decimally diluted in Ringer’s solution, inoculated in MRS and M17, and respectively incubated at 37 °C and 30 °C. The acidified MRS agar (aMRS) was prepared according to the manufacturer’s instructions, acidified to 5.4 with glacial acetic acid (Merck, Darmstadt, Germany), and then autoclaved at 121 °C for 20 min.

### 2.9. Culture-Independent Viable Counts of the Co-Cultures 

Fluorescence microscopy count was performed using the LIVE/DEAD^®^ Baclight ^TM^ Bacterial Viability kit (Molecular Probes, OR, USA), as previously reported (18–20). Nikon filter set B2A FITC was used to visualize green-stained cells (SYTO 9) and the Nikon filter set G-2E/C to visualize red-stained cells (propidium iodide). Pictures of each field were taken and then superimposed through the Nis Elements software (ver. 2.10 Nikon Instruments Inc., Walt Whitman Road Melville, NY 11747-3064, USA) [18,19,20]. Cells were counted as previously described [18,19,20]. A minimum of five separate microscopic fields were counted for each sample. Results were reported as average values ± the standard deviation of the total, viable, and non-viable cells referred to 1 mL.

### 2.10. Statistical Analysis

To investigate the effect of the different concentrations of antimicrobics on the strains, a two-way analysis of variance (ANOVA) model was performed using SPSS Statistics v. 25 (IBM, Armonk, NY, USA). One-way ANOVA and Tukey’s HSD post hoc test were applied to test significant differences (*p* < 0.05).

## 3. Results and Discussion

### 3.1. Single Strains Selection

**Figure 2:**
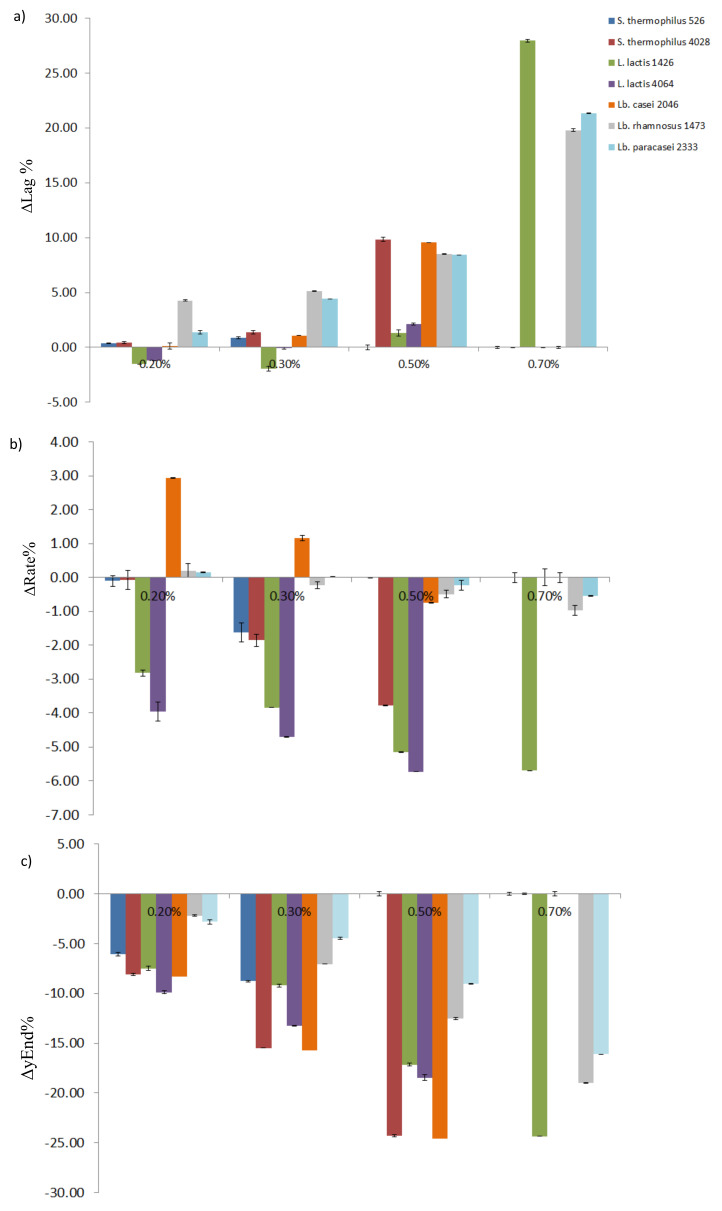
Graphical representation of the Δvalues of each parameter calculated as the difference from the control value 0%. In the figure: (**a**) ΔLag; (**b**) ΔRate; (**c**) ΔyEnd. On the X-axis, the spirulina extract concentrations are reported: 0.20%, 0.30%, 0.50%, and 0.70%.

The impedometric analysis of the single strains cultures was performed by means of BacTrac 4300^®^, which enables the measurement of their growth in real time by registering the variation in electrical conductivity in the medium. In fact, during duplication viable bacterial cells break down sugars present in the medium into the smallest molecules, which increase the conductivity of the medium. Therefore, the quantification of this variation had been used as a measure of the microbial metabolism [15] and, in the present study, as a measure of lactic acid fermentation [17]. The instrument is able to simultaneously measure and record, every 10 min, two impedance values for each single measurement: (i) the *M*-value, which corresponds to the change in the overall medium impedance, and (ii) the *E*-value, which is the measure of the impedance variation in the vicinity of the electrodes. Both these values are shown as relative changes compared to a starting value and expressed as *M*% and *E*% [17]. At the end of the analysis, *M*% values were approximated [15] and used to calculate the kinetic parameters useful to describe the effect of *A. platensis* extract on strain growth. Differently from previous research, where the bactericidal and bacteriostatic effects of *A. platensis* extract were evaluated using a Lag parameter as a measure of cells’ adaptation time and yEnd as a measure of the maximum acidifying capacity [13,15], in this study the Rate parameter was also considered and used as a measure of the acidification rate. The greater the Rate value, the faster the acidification speed of the strain (Table 2). In Table 2, three kinetic parameters are reported for each considered strain and concentration of *A. platensis* extract. Usually, SLAB used for cheese-making are required to rapidly convert lactose into lactic acid, with the consequent fast acidification of the curd [21,22]. Thus, regarding growth parameters, a SLAB strain is expected to have a short Lag phase, a high acidification Rate, and also a good acidifying capacity, showing a high value of yEnd [15]. Both for *S. thermophilus* strains 526 and 4028, the Lag and Rate values in SSM with 0.20% of *A. platensis* extract did not significantly differ from the control (0%) (Table 2). By increasing the *A. platensis* extract concentration in SSM, a significant increase in the Lag parameter was observed. On the other hand, the Rate and yEnd values showed a significant decrease, until any growth was detected for *S. thermophilus* 526 in the presence of 0.50% and 0.70% and *S. thermophilus* 4028 in the presence of 0.70% of *A. platensis* extract (Table 2). These results mean that the increasing concentration of antimicrobial in SSM led to a delay in the growth of both the strains, together with a negative effect on their growth speed and acidifying capacity. Regarding the two other strains belonging to the SLAB species, *L. lactis* 1426 and *L. lactis* 4064, an interesting trend in the growth was found. In fact, the Lag values of both strains grown in the presence of 0.20% and 0.30% of extract were statistically lower or equal compared to the Lag values recorded for the control (0%) (Table 2). In particular, the strain *L. lactis* 1426 showed a significant decrease in the Lag values in the presence of 0.30% of the antimicrobial extract. This could be ascribed to the strain-specific ability to metabolize some components present in the *A. platensis* extract, such as small peptides or monosaccharides derived from the breakdown of extracellular polysaccharides [23]. This trend was observed only for the Lag values, while other considered parameters, Rate and yEnd, were gradually decreased by increasing the concentration of antimicrobial in the SSM (Table 2). On the other hand, NSLAB are expected to show a slow growth as well as low acidification Rate and scarce acidifying capacity, as their role has to be played after the acidification, in particular during ripening [3,24,25]. Based on this, NSLAB are supposed to have a long Lag time, with small values for Rate and yEnd. In the present study, despite belonging to a species classified as NSLAB [21], *Lb. casei* 2046 showed behavior that was comparable to that of the SLAB species. Indeed, it had a short Lag phase and a good acidification capacity when grown in SSM with 0.20% of *A. platensis* extract. As observed for SLAB, increasing concentrations of the extract had higher impacts on the growth parameters (Table 2). The other NSLAB strains, *Lb. rhamnosus* 1473 and *Lb. paracasei* 2333, showed different responses to the *A. platensis* extract as compared to *Lb. casei* 2046. Actually, they had a statistically longer Lag phase as compared to the control at each antimicrobial concentration considered. Furthermore, increasing concentrations of the extract in SSM also affected the acidification capacity of both the strains, as can be observed in the decreasing yEnd recorded values (Table 2). For a better view of the effect of *A. platensis* extract on the strains’ growth, a graphical representation of the Δ value of each parameter, calculated as the difference with respect to the control value (0%), was reported (Figure 2). For all the parameters (ΔLag, ΔRate, and Δyend), the tallest histogram bars, the highest increase or decrease in the values were observed (Figure 2a). Taken together, the data show that the *A. platensis* extract concentration of 0.30% did not interfere with the starter activity. On the other hand, it was responsible for the delay in the NSLAB growth (Figure 2). For this reason, this concentration was chosen to be tested in the next experimental step. As the SLAB strain *L. lactis* 1426 was stimulated by the presence of this amount of *A. platensis* extract in SSM with a decrease in ΔLag of 1.92 h (Figure 2), it was considered as the most suitable candidate to be used as a starter in the in vitro simulation of co-culturing. In the same way, the NSLAB strain *Lb. rhamnosus* 1473 showed a good increase in the Lag phase as compared to the control and a good reduction in the acidifying Rate and capacity when grown in SSM with 0.30% of *A. platensis* extract, thus it was chosen to be used as NSLAB in the in vitro simulation of co-culturing. This choice was corroborated by the fact that the lactobacilli isolated by quality cheeses, as in the case of *Lb. rhamnosus* 1473, showed a positive effect on cheese quality during ripening [11].

### 3.2. In Vitro Simulation of SLAB L. lactis 1426 and NSLAB Lb. rhamnosus 1473 Co-Culturing

**Figure 3:**
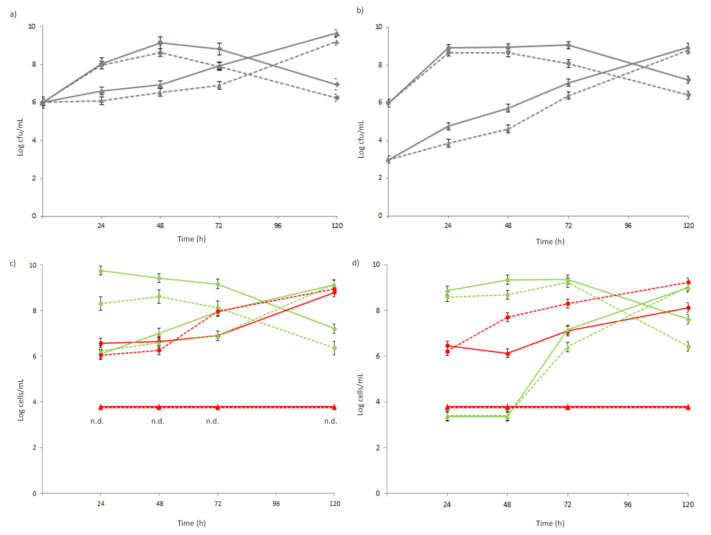
Enumeration of *L. lactis* 1426 (●) and *Lb. rhamnosus* 1473 (▲) cultivated in co-culture for 120 h in the presence (dotted lines) and absence (solid line) of 0.30% *A. platensis* extract. Plate counts of co-culture 1 (**a**) and co-culture 2 (**b**). Fluorescent microscopic counts of co-culture 1 (**c**) and co-culture 2 (**d**): the number of viable cellsare in green and in red are the number of dead cells (n.d.: not detected; the cells’ concentration was under the 3 Log cells/mL).

Two co-coltures were prepared with the chosen strains. Co-culture 1 was obtained by mixing *L. lactis* 1426 and *Lb. rhamnosus* 1473 in SSM in a ratio of 1:1, while co-culture 2 was obtained by mixing *L. lactis* 1426 and *Lb. rhamnosus* 1473 in SSM in a ratio of 1000:1 to simulate the proportion commonly used in cheese making processes [26]. The growth dynamics of the two strains co-cultured for 120 h were monitored by three different approaches: (i) plate counting on M17 for the enumeration of lactococci and MRS for lactobacilli; (ii) the fluorescent microscopic counting of viable (green) and non-viable (red) coccoid cells of *L. lactis* 1426 and rod-shaped cells of *Lb. rhamnosus* 1473; (iii) the overall estimation of co-culture growth behavior by impedometric parameters Lag, Rate, and yEnd. The initial concentrations of the strains in each co-culture were confirmed by plate count, as reported in (Figure 3a,b).

**Figure 4:**
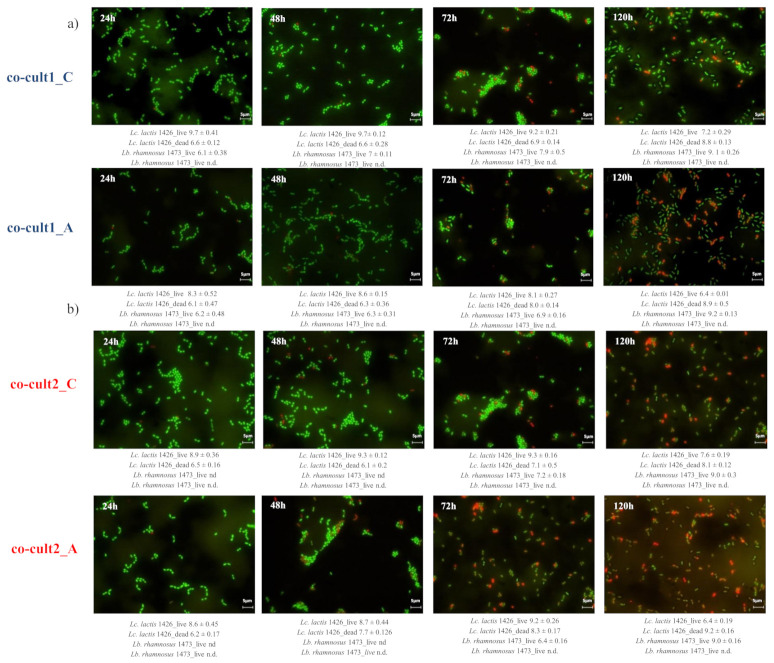
Fluorescence microscopy images of co-culture 1 (**a**) and co-culture 2 (**b**), with (co-Cult_A) and without (co-cult_C) 0.3% of antimicrobial *A. platensis* extract at four sampling time points: 24, 48, 72, and 120 h of incubation. Live cells labelled with Syto 9 appear in green, and dead cells labelled with propidium iodide (PI) appear in red. Scale bars are 5 μm in length. The Starter strain cells Lc. 1426 are round-shaped, while the non starter strain cells Lb. 1473 are rod-shaped.

The growth of the starter strain *L*. *lactis* 1426 was not affected by the presence of *A. platensis* extract in either the co-cultures showing a very similar trend to the control. Only after 48 h of fermentation was a progression of natural cells mortality observed for the starter strain *L*. *lactis* 1426 in both the co-cultures (Figure 3a,b). Conversely, the NSLAB strain *Lb. rhamnosus* 1473 kept growing during the 120 h of incubation while showing a significant slowest growth and a lowest cell concentration between 24 and 72 h (Figure 3a,b) in the presence of the *A. platensis* extract. These results were confirmed by fluorescence microscopy, which allowed us to count both live and dead cells (Figure 3c,d for co-culture 1 and 2, respectively). However, it has to be noted that cell concentrations under 4 Log cells/mL were not detected (nd) with this method; thus, the level of viable and dead cells in (Figure 3c,d) under this level could be due to the detection limit. At first, the behavior of the single strains composing the two co-cultures was evaluated. The number of live cells of *L. lactis* 1426 in co-culture 1 was always significantly higher, up to 1 Log, in the control than in the presence of the antimicrobial extract (Figure 3c). These differences can be clearly observed at each sampling point of (Figure 4a) co-cult1_C, where the green coccoid cells were always predominant. Conversely, in the presence of *A. platensis* extract, the number of dead cells increased progressively, up to approximately 9 Log cells/mL at 120 h (Figure 3b and red coccoid cells in Figure 4a co-cult1_A). This could be attributable to the antimicrobial effect of the extract on the cells. In particular, the effect could be ascribed to the presence of ϒ-linoleic acid, 1-acetodecane, and especially high lipid concentrations, typical of spirulina extract, which could have compromised the integrity of the cellular membrane [27]. Although the initial concentration of the inoculated cells was the same in both the co-cultures, the behavior observed for the starter strain *L. lactis* 1426 in co-culture 2 was different. In fact, the number of live cells was constantly lower in the presence of *A. platensis* extract until 72 h of incubation, when the maximum amount of live cells was detected in both the conditions. After that, the live cell count decreased down to the minimum value at 120 h (Figure 3d and Figure 4b, co-cult2_C and co-cult2_A). In parallel, the dead *L. lactis* 1426 cells count was always higher in the presence of the antimicrobial, until the end of fermentation (120 h) (Figure 3d and red coccoid cells in Figure 4b, co-cult2_C and co-cult2_A).

Concerning the NSLAB strain *Lb. rhamnosus* 1473, the number of live cells in co-culture 1 was similar at the beginning of fermentation and after 24 and 120 h of incubation, while it showed a slower growth in the presence of antimicrobial extract between 24 and 48 h (Figure 3c and green rod-shaped cells in Figure 4a co-cult1_C and co-cult1_A). Differently, the number of live *Lb. rhamnosus* 1473 cells in co-culture 2 was under the limit of detection for the first 48 h, as expected. In both co-culture 1 and 2 (Figure 3c,d) the number of dead *Lb. rhamnosus* 1473 cells was always under the limit of detection. Despite different initial cell concentrations, in both the co-cultures the presence of *A. platensis* extract slowed down the growth of NSLAB compared to the control (Figure 3d; Figure 4b co-cult2_C and co-cult2_A green rod-shaped cells).

This means that the adjunct culture needed more time to reach the maximum live cell concentration. This has two important effects: (i) given that the cells need more time to grow, they might not interfere with the SLAB activity in the first hours of fermentation, thus avoiding one of the main drawbacks of using secondary adjunt cultures; (ii) the cells could stay alive in the fermented product for a longer period. This is also in good agreement with the already-observed effect of Spirulina in preserving the viability of some probiotic cultures [28].

The overall behavior of the two strains, observed by means of Bactrac 4300^®^ in both co-cultures, showed significantly lower values for all the impedometric parameters in the presence of the antimicrobial (Table 3). In particular, for the strain *L. lactis* 1426, lower Lag values were observed in the presence of *A. platensis* extract. This interesting stimulatory effect, observed only for this strain, could be ascribable to the presence of carbohydrates, free AA, and proteins deriving from the extraction process [29], or to the presence of polysaccharides from spirulina cell breakdown, whose boosting effect on LAB growth has been already reported by Golmakani (et al., 2019) and Martelli (et al., 2020) [28,30,31]. The presence of the extract also affected the Rate values. In fact, in the presence of the antimicrobial, the Rate values were lower, which means a slower cell duplication time. Conversely, the overall acidification capacity was lower in the presence of the antimicrobial, despite an initial stimulatory effect being observed (Table 3). 

Concerning the pH values measured at each sampling point no significative differences were found among the co-cultures, except for both co-culture 1 and 2, which in the presence of the extract showed significantly lower values for pH. This support the results obtained with the impedometric analysis, where a slowing down of the growth and overall acidification capacity was observed.

## 4. Conclusions

The use of *A. platensis* extract, which, differently from dried Spirulina powder. has neither an unpleasant odor nor color, has been found to have both stimulatory and inhibitory effects on different LAB strains. In particular, we observed a very interesting behavior of one SLAB strain grown in co-culture with a NSLAB strain in the presence of 0.3% of antimicrobial. The growth of the NSLAB strain *Lb. rhamnosus* 1473 was slowed down by the antimicrobial’s presence, while, on the contrary, the LABSLAB strain *L. lactis* 1426 was not negatively affected. Furthermore, the growth of the NSLAB strain was delayed without compromising the cells’ integrity, meaning that the metabolic potential of these cells is preserved. This can be of great interest for a possible application of attenuated NSLAB in the formulation of adjunct cultures to be employed, for example, in the cheesemaking process.

Despite the fact these results have to be considered as preliminary and further in vivo experiments are needed, it is possible to conclude that the used approach, by means of impedometric analysis, could be successfully applied in the screening of other natural antimicrobials to be used to attenuate the growth of adjunct cultures.

## Figures and Tables

**Table 1:** Strains, origin and growth conditions.

Species	Strain	Origin	Growth Conditions	Reference Paper
*Streptococcus thermophilus*	526	Milk	M17, 42 °C anaerobiosis	[15]
*Streptococcus thermophilus*	4028	Curd	M17, 42 °C anaerobiosis	[15]
*Lactococcus lactis*	4064	Cheese	M17, 30 °C anaerobiosis	[15]
*Lactococcus lactis*	1426	Milk	M17, 30 °C anaerobiosis	[15]
*Lacticaseibacillus casei*	2046	Cheese	MRS, 37 °C anaerobiosis	[16]
*Lacticaseibacillus paracasei*	2333	Cheese	MRS, 37 °C anaerobiosis	[16]
*Lacticaseibacillus rhamnosus*	1473	Cheese	MRS, 37 °C anaerobiosis	[16]

**Table 2:** Lag, Rate, and yEnd parameters of the seven considered strains grown in skimmed milk (SSM) with different *A. platensis* extract concentrations.

Strains		*A. platensis* Extract Concentration				
		0%	0.20%	0.30%	0.50%	0.70%
*S. thermophilus 526*	Lag	1.03 ^b^ ± 0.01	1.39 ^b^ ± 0.08	1.91 ^a^ ± 0.21	nd ^c^ ± 0.11	nd ^c^ ± 0.18
	Rate	5.40 ^a^ ± 0.15	5.30 ^a^ ± 0.28	3.77 ^b^ ± 0.01	nd ^c^ ± 0.01	nd ^c^ ± 0.01
	yEnd	29.35 ^a^ ± 0.18	23.29 ^b^ ± 0.08	20.59 ^c^ ± 0.20	nd ^d^ ± 0.16	nd ^d^ ± 0.01
*S. thermophilus 4028*	Lag	<1 ^c^ ± 0.09	1.41 ^c^ ± 0.14	2.37 ^b^ ± 0.19	10.84 ^a^ ± 0.01	nd ^d^ ± 0.01
	Rate	4.06 ^a^ ± 0.28	3.98 ^a^ ± 0.18	2.2 ^b^ ± 0.01	0.29 ^c^ ± 0.15	nd ^c^ ± 0.01
	yEnd	29.27 ^a^ ± 0.11	21.20 ^b^ ± 0.01	13.79 ^c^ ± 0.08	4.97 ^d^ ± 0.07	nd ^e^ ± 0.01
*L. lactis 1426*	Lag	4.95 ^b^ ± 0.01	3.42 ^c^ ± 0.22	3.01 ^c^ ± 0.28	6.24a ± 0.12	32.93 ^d^ ± 0.08
	Rate	5.79 ^a^ ± 0.08	2.97 ^b^ ± 0.01	1.95 ^c^ ± 0.01	0.64 ^d^ ± 0.01	0.09 ^e^ ± 0.01
	yEnd	27.39 ^a^ ± 0.21	19.90 ^b^ ± 0.12	18.18 ^c^ ± 0.15	10.23 ^d^ ± 0.01	3.06 ^e^ ± 0.28
*L. lactis 4064*	Lag	3.95 ^b^ ± 0.01	2.74 ^c^ ± 0.08	3.87 ^b^ ± 0.08	6.07 ^a^ ± 0.01	nd ^d^ ± 0.01
	Rate	6.11 ^a^ ± 0.28	2.15 ^b^ ± 0.01	1.41 ^c^ ± 0.01	0.39 ^d^ ± 0.25	nd ^e^ ± 0.08
	yEnd	27.05 ^a^ ± 0.15	17.17 ^b^ ± 0.02	13.80 ^c^ ± 0.28	8.6 ^d^ ± 0.21	nd ^e^ ± 0.01
*Lb. casei 2046*	Lag	1.28 ^b^ ± 0.26	1.41 ^b^ ± 0.01	2.37 ^c^ ± 0.06	10.84 ^a^ ± 0.01	nd ^d^ ± 0.08
	Rate	1.04 ^c^ ± 0.01	3.98 ^a^ ± 0.08	2.21 ^b^ ± 0.01	0.29 ^d^ ± 0.15	nd ^e^ ± 0.01
	yEnd	29.52 ^a^ ± 0.13	21.20 ^b^ ± 0.11	13.79 ^c^ ± 0.07	4.97 ^d^ ± 0.08	nd ^e^ ± 0.01
*Lb. rhamnosus 1473*	Lag	8.43 ^e^ ± 0.15	12.72 ^d^ ± 0.01	13.55 ^c^ ± 0.06	16.94 ^b^ ± 0.01	28.25 ^a^ ± 0.01
	Rate	1.19 ^a^ ± 0.21	1.40 ^a^ ± 0.1	0.96 ^b^ ± 0.11	0.70 ^c^ ± 0.15	0.22 ^d^ ± 0.12
	yEnd	22.01 ^a^ ± 0.08	19.81 ^b^ ± 0.01	14.96 ^c^ ± 0.14	9.49 ^d^ ± 0.01	3.02 ^e^ ± 0.01
*Lb. paracasei 2333*	Lag	10.32 ^e^ ± 0.08	11.71 ^d^ ± 0.02	14.73 ^c^ ± 0.01	18.74 ^b^ ± 0.13	31.67 ^a^ ± 0.01
	Rate	0.69 ^b^ ± 0.01	0.84 ^a^ ± 0.01	0.71 ^b^ ± 0.15	0.47 ^c^ ± 0.12	0.15 ^d^ ± 0.28
	yEnd	19.69 ^a^ ± 0.21	16.89 ^b^ ± 0.11	15.24 ^c^ ± 0.03	10.69 ^d^ ± 0.01	3.60 ^e^ ± 0.01

Lag is reported in hours; nd: not detected within 48 h. Values are the mean ± SD of at least two separate experiments in which each variable was measured in triplicate. Different superscript lowercase letters a–e within the same row highlight significant differences according to ANOVA (*p* < 0.05) among each parameter at different *A. platensis* concentrations.

**Table 3:** Impedometric parameters describing the growth of the 2 co-cultures with (A) and without antimicrobial *A. platensis* extract (C).

	Co-Culture1_C	Co-Culture1_A	Co-Culture2_C	Co-Culture2_A
Lag	6.29 ± 0.02 ^a^	5.79 ± 0.08 ^b^	6.60 ± 0.06 ^a^	5.85 ± 0.03 ^b^
Rate	4.94 ± 0.01 ^a^	1.53 ± 0.02 ^b^	5.00 ± 0.05 ^a^	1.16 ± 0.04 ^b^
yEnd	26.71 ± 0.06 ^a^	15.44 ± 0.05 ^b^	25.95 ± 0.08 ^a^	13.78 ± 0.07 ^b^

Values are the mean ± SD of at least two separate experiments in which each variable was measured in triplicate. Different superscript lowercase letters within the same raw highlight significant differences according to ANOVA (*p* < 0.05) among each parameter.

## Data Availability

Not applicable.
